# Cognitive Interventions in Older Persons: Do They Change the Functioning of the Brain?

**DOI:** 10.1155/2015/438908

**Published:** 2015-10-25

**Authors:** Yindee van Os, Marjolein E. de Vugt, Martin van Boxtel

**Affiliations:** ^1^Department of Medical Psychology, Elkerliek Hospital, Wesselmanlaan 25, 5507 HA Helmond, Netherlands; ^2^School for Mental Health and Neuroscience, Alzheimer Centre Limburg, Department of Psychiatry and Neuropsychology, Maastricht University, P.O. Box 616, 6200 MD Maastricht, Netherlands

## Abstract

*Background*. Cognitive interventions for older persons that may diminish the burden of cognitive problems and could delay conversion to dementia are of great importance. The underlying mechanisms of such interventions might be psychological compensation and neuronal plasticity. This review provides an overview of the literature concerning the evidence that cognitive interventions cause brain activation changes, even in damaged neural systems. *Method*. A systematic search of the literature was conducted in several international databases, Medline, Embase, Cinahl, Cochrane, and Psychinfo. The methodological quality was assessed according to the guidelines of the Dutch Institute for Health Care Improvement (CBO). *Results*. Nineteen relevant articles were included with varied methodological quality. All studies were conducted in diverse populations from healthy elderly to patients with dementia and show changes in brain activation after intervention. *Conclusions*. The results thus far show that cognitive interventions cause changes in brain activation patterns. The exact interpretation of these neurobiological changes remains unclear. More study is needed to understand the extent to which cognitive interventions are effective to delay conversion to dementia. Future studies should more explicitly try to relate clinically significant improvement to changes in brain activation. Long-term follow-up data are necessary to evaluate the stability of the effects.

## 1. Introduction

Aging is accompanied by changes in our cognitive functioning based on structural and functional changes in the brain [1, 2]. Many older persons complain about diminished cognitive functioning [3]. Cognitive complaints and cognitive deficits are often a burden for older persons and their family [4–6]. Cognitive deficits could also be a precursor for dementia. In that case it is important to intervene in an early stage to prevent or delay conversion to dementia and to minimize the impact of these objective or perceived cognitive problems [5, 7].

Pharmacological interventions are of limited efficacy, may have serious side effects, and are only available for patients with a clinical diagnosis of dementia [8]. Cognitive interventions have gained a lot of attention over the last years. The main goal of cognitive interventions is to stimulate the cognitive system or offer compensatory methods to address difficulties with cognitive functioning [7, 9]. Clinicians acknowledge the benefits of cognitive interventions such as changing a patient's sets of beliefs, affective states, or behavioural patterns and compensating for cognitive losses [10].

A literature review performed by Buschert and colleagues in 2010 highlights the effectiveness of cognitive interventions in improving global cognitive functioning, daily activities, quality of life, and diminishing behavioural problems in patients with Mild Cognitive Impairment (MCI) or dementia [8]. A review of randomized controlled trials on this topic in persons with MCI concluded that there is evidence for intervention success in overall cognition, overall self-ratings, episodic memory, and executive function/working memory. The quality of the evidence is limited due to several methodological issues such as a small amount of long-term follow-up measures with generally small effect sizes. Moreover there are differences in design, sample sizes, and types of intervention [11].

Cognitive stimulation in a social setting such as reminiscence with reality orientation is associated with benefits in cognitive functioning as well as quality of life, well-being, communication, and social interaction skills [9]. In healthy elderly, cognitive interventions lead to fewer negative emotional reactions towards cognitive functioning [3, 12, 13], improvement in coping with reported cognitive failures [12, 13], and better objective cognitive functioning [13, 14]. Another important goal of cognitive interventions is intervening at an early stage of cognitive decline to slow or prevent progression to dementia. Cognitive interventions could even have the potential to delay the onset of Alzheimer's Dementia (AD) by 5 years in those patients at risk for AD [8]. The evidence for the efficacy of cognitive interventions is promising yet inconclusive due to differences in design, outcome measures, interventions, and sample sizes. The low costs, absence of adverse effects, and the possibility to delay the onset of Alzheimer's dementia make cognitive interventions attractive [8]. Some even stated that neurobiological outcomes might be used as a sensitive biomarker for the efficacy of cognitive training [15].

The mechanisms underlying the effectiveness of cognitive interventions are not well understood. A better understanding of these mechanisms can help us tailor our cognitive interventions and possibly improve the effectiveness.

One mechanism might be psychological compensation. Cognitive interventions would then improve coping strategies to deal with the still existing cognitive problems [3]. In clinical practice, the working mechanism and goals of cognitive interventions are explained to participants in terms of this psychological compensation. But recent neural models suggest that neuronal plasticity may also underlie the effectiveness of cognitive interventions [5, 8, 16–18]. Cognitive interventions might increase cognitive reserve that is reflected in changes in brain activation patterns [4, 8]. Cognitive reserve, the capacity of an adult brain to cope with brain pathology in order to minimize symptomatology, is linked to efficiency (less activation of brain networks) in healthy elderly. On the other hand, in pathological aging, cognitive reserve enhances the recruitment of compensating brain networks particularly the frontal areas, hippocampus, and the precentral gyrus [1, 19].

*The aim* of this paper is to review the evidence that cognitive interventions cause brain activity changes. Eventually, early intervention in prodromal stages to delay conversion to pathological aging is the ultimate goal. Therefore the effects of cognitive interventions on brain activity changes are studied in the brain of healthy older persons and in the brain of older persons suffering from pathological aging. To relate changes in brain activation to intervention effect, evidence of changes in performance, function, behaviour, and cognition are also reviewed.

## 2. Methods

### 2.1. Search Strategy

A systematic literature search of articles published from 1993 to March 2012 was conducted in Medline, Embase, Cinahl, Psychinfo, and the Cochrane Database. Medical Subject Headings (MeSH) terms, thesaurus terms, and an age selection > 60 years were used in the search (search terms used in selection of studies) as follows:

1. Psychinfo: cognitive impairment OR dementia OR age selection set on > 60 years, Medline and Cinahl: cognition disorder OR dementia OR aged OR elderly, and Embase: cognitive defect OR dementia OR aged OR elderly;
2. Psychinfo: behavioural therapy OR cognitive therapy Medline, Cinahl, Embase: behavior therapy OR cognitive therapy;
3. Psychinfo: magnetic resonance imaging OR tomography OR electrophysiology, Medline and Cinahl: magnetic resonance imaging OR electroencephalography OR tomography, and Embase: nuclear magnetic resonance imaging OR electroencephalogram OR tomography.

**Figure 1:**
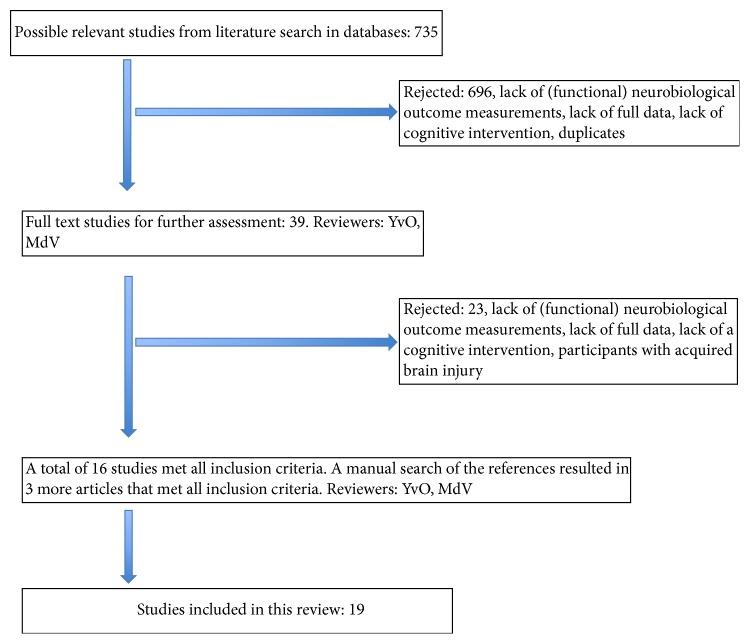
Flowchart of the search strategy.

Reference lists of the retrieved studies were searched to identify any relevant articles that had not yet been included. To be selected for this review, papers had to meet the following criteria: (i) the study had to involve the healthy elderly, the healthy elderly with cognitive complaints, and the elderly with Mild Cognitive Impairment or elderly with dementia; (ii) the study had to contain a cognitive intervention; (iii) the study had comparisons of brain activity measurements before and after the intervention; (iv) the study had a full report available; (v) the articles were in English. A flowchart of the inclusion process is shown in Figure 1.

### 2.2. Selection of Studies

The search resulted in 735 papers. One reviewer (YvO) screened all titles and abstract for suitability. Six hundred ninety-six studies were rejected because of duplication, lack of full data, lack of brain activity measures, or lack of a cognitive intervention. The remaining 39 studies were obtained in full text and assessed by two reviewers (YvO, MdV). There was full agreement on the exclusion of 23 papers because they did not meet all inclusion criteria. A manual search of the reference lists of the included studies resulted in 3 additional papers. A total of 19 studies met all inclusion criteria. Figure 1 shows the flowchart of the selection process of the papers.

### 2.3. Methodological Quality

The methodological quality of the included studies was assessed according to the guidelines of the Dutch Institute for Health Care Improvement (CBO). The CBO aims to improve healthcare by providing guidelines for evidence-based interventions both nationally and internationally (CBO, http://www.cbo.nl/). For randomized controlled trials, the following aspects were evaluated: randomization, blinding of randomization, blinding of participants, blinding of outcome assessors, baseline comparability, loss to follow, use of intent-to-treat analysis, comparability of intervention, and a judgment of the validity and applicability of the study. Because it is impossible in cognitive intervention trials to blind the therapists to the intervention, this item was not included as one of the quality criteria. With regard to studies that did not contain a randomized controlled design, the following aspects were evaluated: definition of study population, selection bias, intervention description, outcome definition, blinded outcome assessments, completeness of the dataset/follow-up, loss to follow, confounders, and a judgment of the validity and applicability of the study.

Eventually, the overall quality of the individual studies was rated in a level of evidence, ranging from A1 to D. A1 is a systematic review of at least two independent, randomized double-blind studies of sufficient quality and size. A2 is a randomized double blind study of sufficient quality and size. B is a comparative study, which does not meet all the criteria of an A2 study. C is a noncomparative study and D is the opinion of experts (http://www.cbo.nl/). Two reviewers (YvO and MdV) independently assessed the methodological quality of the studies. The level of agreement was 96%. After a consensus meeting, both reviewers reached full agreement on the quality ratings. The quality ratings of the studies are displayed in Tables 1 and 2.

## 3. Results

### 3.1. The Healthy Elderly

#### 3.1.1. Study Characteristics

Six studies focused on healthy older adults [18, 20–23, 30]. Two studies investigated the effect of the method of loci, a mnemonic technique, on brain activation [18, 30]. The intervention duration varied between these two studies from one day [30] to five weeks [18]. The neurobiological outcomes were different; the one-day intervention used Positron Emission Tomography (PET) measures and a memory test as outcomes [30]. The other study used magnetic resonance imaging (MRI), Magnetic Resonance Spectroscopy (MRS), performance on a memory test, and level of depression and anxiety as outcomes. One study lacked a control group [30], and the other study had a randomized controlled trial (RCT) design with a small sample size.

A third study [20] used a RCT design and studied the effect of a multicomponent intervention in 17 older persons with mild subjective memory complaints. Eight of them took part in the intervention that consisted of a diet, relaxation exercises, brain-teasers, memory strategies, and cardiovascular physical training. The PET and MRI data, performance on cognitive tasks, and scores on the subscale self-awareness of memory ability of the Mood and Feelings Questionnaire (MFQ) were collected.

A fourth study [22] evaluated the benefits of 25 sessions of computerized working memory training on neuropsychological tests varying in level of similarity to those practiced in the training. FMRI measures were studied to evaluate differences in brain activity post intervention. The strength of this study is the use of an active control group that also received a working memory training but with a fixed low level task load. The intervention group received an adaptive training with increasing task load.

Two studies [21, 23] studied the effect of repeated practice with performance feedback on brain activity. Both used single and dual tasks to study focused and divided attention. Belleville and collaborators [23] added a condition in which participants were instructed on the modulation of their attention to the tasks. They were interested if the training format would influence brain activity patterns after intervention. To investigate this hypothesis, all 48 community dwelling older adults were randomly assigned to one of the three intervention conditions, for example, single task, dual task, or top down control of attention. In the other study the 34 older participants were randomly assigned to either a waiting list or the intervention.

The intervention consists of 5 [21] or 6 [23] sessions. Both used fMRI and MRI as neurobiological outcomes. The reaction time and accuracy of the task performances were collected and served in both studies as behavioural data. Moreover both studies statistically examined the relation between performance gains and brain activity changes after intervention.

#### 3.1.2. Findings

The six studies differ in the type of intervention, sample size, design, neurobiological outcomes, and the presence of more clinically relevant outcomes to measure intervention success (Table 2). All* six* studies found brain activity differences after intervention.

Participants in the study of Valenzuela et al. learned the same mnemonic (method of loci). They showed increases in brain activation in the hippocampus [18] and the left occipital parietal cortex and left retrosplenial cortex [30]. These areas are known to be associated with the cognitive domains that were targeted in the intervention. Post hoc relation between performance improvement and brain activity changes was identified in the Nyberg study. Only the older adults who also improved on a memory test showed these brain activity increases [30].

The study of Small and colleagues (2006) demonstrated decrease in the dorsolateral brain activation after intervention and an improvement in verbal fluency. There was no significant intervention effect on subjective measures of self-awareness of memory performance and a memory task [20].

There are three studies that explicitly tried to link intervention related performance gains to brain activation changes. Participants that profit the most from an adaptive working memory training showed the largest activation decreases in regions known to be involved in memory and attention processes (e.g., right inferior frontal, right inferior parietal, left fusiform region, and insula) and the largest activation increases in the caudate [22]. Another study provided evidence that training induced dual task performance improvement was related to increased activity in the left ventral prefrontal cortex and decreased activity in the dorsolateral prefrontal cortex. Compared with an adult sample, age related differences in brain activity were reduced after intervention [21]. The study of Belleville and colleagues [23] found significant correlations between performance and training related activity. They provide evidence that type of intervention influenced the loci and type of brain activity. Repeated practice in single tasking was associated with a decreased activity in the inferior and middle frontal gyri bilaterally and the left thalamus. The computed correlations revealed that a better performance in single tasking was correlated with decreased activity in the right inferior and middle frontal gyrus. Repeated training in dual tasking resulted in greater activity in the prefrontal cortex during dual tasking. There were no significant correlations between performance gains and brain activity changes for this condition. For the strategic control of attention condition, dependent on the type of instruction, increased activity after training in the right middle frontal gyrus or the right cerebellum was seen. An improved ability to modulate attention according to task instruction was correlated with a greater activity in Brodmann area 10 [23].

However, how the brain activity changes in all studies were related to clinical relevant improvement is not clear, due to a lack of such outcomes and a fail to link brain activation changes to clinically meaningful improvement [18, 20–23, 30]. The stability of the intervention effects was unclear and long-term follow-up measures were lacking [18, 20–23, 30].

### 3.2. Patients with MCI

#### 3.2.1. Study Characteristics

All five studies [16, 24, 31–33] that focused on MCI patients used fMRI to investigate the influence of a cognitive intervention on brain activity. However the targets of the cognitive intervention as well as the design of the studies differed.

The most recent study with an RCT design evaluated the effects of a mnemonic strategy training on object location associations and brain activation in the hippocampus via fMRI. A matched control group was also exposed to the same lists of object location associations, but without learning the mnemonic. Eighteen participants had a diagnosis of amnestic MCI. A group of 16 healthy controls were allocated to the same treatment conditions. Both diagnostic groups were comparable in terms of prognostic factors, and intervention success was evaluated by a modified change score for learning object location associations [32].

One study used an episodic memory training of 6 weeks (mnemonics and psychoeducation) with proven effectiveness for a MCI population. Fifteen participants with the diagnosis amnestic MCI and 15 matched healthy controls took part in the intervention. The data on visual memory, MRI, and fMRI were collected at baseline and after intervention. Brain activity was studied during encoding and retrieval of a memory test. A double baseline was used to study the possibility of a repetition effect. Furthermore preexisting brain activation differences between both groups were investigated by comparing fMRI data at baseline [33].

Another study [16] randomly assigned 12 MCI patients to a cognitive intervention or an active control group. The computer-based cognitive intervention aimed to improve processing speed and accuracy of auditory processing. It was a time consuming intervention. Participants used the cognitive training five days a week for a hundred minutes per day for two months. The active control group made computer-based exercises with comparable time intensity. Pre- and postintervention fMRI data and a memory test were administered [16].

A face-name association learning task was used as an intervention in an earlier study of Hampstead and colleagues [32]. They studied the effect of this face-name association learning in six amnestic multidomain MCI patients. The fMRI was administered before and after the 5 training sessions. They compared fMRI results of trained with untrained lists in different sessions to compensate for repetition effects of fMRI. Furthermore they computed a functional connectivity analysis [32].

In a single case design, the effect of a goal oriented cognitive rehabilitation was studied in a person with amnestic MCI. Besides fMRI data, performance on a memory tests and an anxiety and depression questionnaire were collected. Finally, the progress in personal rehabilitation goals was evaluated. A face-name association learning task was administered during the fMRI [31].

#### 3.2.2. Findings

Despite the differences in methodologies, types of intervention, and sample sizes in the studies (see Table 3), all investigations found evidence for neurobiological changes in brain activation after intervention.

In an RCT study with an active control group, an intervention related increase in brain activity in the hippocampus was seen, whereas the active control group showed a decreased activity in the hippocampus. Participants that underwent the auditory processing training also improved on a memory test and the training tasks [16].

The other RCT study that evaluated a mnemonic training, found evidence for an intervention related increased activity in the left hippocampal body and the right hippocampus during retrieval of the trained stimuli. For retrieval of the untrained stimuli there was an intervention related increased activity in the right hippocampal. Performance improved after training for the trained stimuli, not for untrained stimuli [24].

In an earlier multiple cases study by the same author, increased connectivity and increased activity in frontal, parietal, temporoparietal areas and precuneus were seen after a face-name association learning training. The six participants also improved on trained and untrained memory tasks [32].

A study that evaluated the effect of an episodic memory training found increased activity in frontal, temporal, and parietal areas. Some areas were already active at baseline; other areas were new, alternative areas. Only increased activity in the right inferior parietal lobe correlated with improvement on a memory test [33].

A single case study found evidence for a pattern of brain activity increases and decreases after a goal oriented cognitive training. Decreases were seen in sensory areas during both encoding and retrieval, such as the higher visual areas, left fusiform gyrus, left medial occipital gyrus. Increased activity was seen in frontal areas, temporoparietal junction, parahippocampal gyrus, and right globus pallidus. Both subjective memory satisfaction and subjective memory performance improved after the intervention [31].

In conclusion, mostly an increase in brain activation specifically in areas typically involved in intervention related cognitive processes [16, 24, 31, 33] was seen, as well as activation of the default network [24, 32]. In several studies, the authors claimed that normalisation of the pattern of brain activation after intervention had occurred as a possible result of restoration [24, 31–33] whereas the activation of additional areas was interpreted as a compensatory mechanism [24, 32].

Most of the studies tried to relate the changes in brain activation to intervention success. Most studies formulate this intervention success as performance improvement on a cognitive task that was the target of the intervention. One single case study also studied subjective complaints of cognition, mood, and anxiety. These authors reported improvement in subjective measures of memory performance and satisfaction [31].

### 3.3. Patients with Dementia

#### 3.3.1. Study Characteristics

Eight studies [25–29, 34–36] focused on patients with dementia. Four of the studies used a RCT design [25–27, 29]. Study population, intervention targets, and outcome measures differed.

The most recent RCT study [29] aimed to improve cognition, behaviour, and motor functioning with an intense, multidimensional stimulation program. The effectiveness of this program was studied in 60 persons with AD on a questionnaire for behavioural and psychiatric problems (NPI), language, and memory scales of the Alzheimer's Disease Assessment Scale-Cognitive subscale (ADAS-cog), functional status, physical well-being, and fMRI. The intervention group was compared to a waiting list. Twenty healthy controls served as a reference for the typical activation pattern while performing the in-scanner verbal fluency task. The strength of this study was a long-term follow-up measurement [29].

In another RCT study [27] the effect of a six-month cognitive intervention program designed to improve global cognitive functioning, mood, and quality of life in patients with MCI and mild AD was investigated. To study disease-related brain activation differences at baseline, PET data of the MCI and AD group were compared with PET data of a group of eleven healthy elderly. In the active control condition, participants made pencil and paper assignments focused on sustained attention (mostly intact in MCI and AD). A specific PET method was used that is known to be more sensitive in detecting disease related metabolic disturbances in mild to moderate stages of neurodegenerative diseases [27].

The RCT study of Heiss et al. [25] also used a six-month intervention for 80 participants with AD. The content of their intervention was different. The 17 participants received social support (group 1), 18 participants received cognitive training twice a week (group 2), 18 participants received a combination of the cognitive training and the drug pyritinol that is used for symptomatic treatment of AD (group 3), and finally 18 participants received cognitive training and the dietary supplement phosphatidylserine. At baseline and at several times during intervention and postintervention neuropsychological tasks, the quantitative EEG, resting state PET, and stimulation PET were administered [25].

The fourth RCT study [26] investigated 24 residents of a geriatric nursing home with a diagnosis of vascular dementia (VaD). They were randomly assigned to reminiscence with reality approach condition or were treated to the standard of care. The duration of the intervention was 3 months. Reminiscence with reality approach claimed to invoke memories with the aid of materials and generated a better awareness of the “here and now.” Scores on a cognitive screening test (MMSE), a mood questionnaire (GDS), a behavioural observational questionnaire (BRSE), and PET data were all collected. A region of interest analysis (anterior cingulate left and right) was conducted with the PET data. [26].

The efficacy of tailored cognitive rehabilitation in 19 people with early stage AD or mixed AD/VaD was evaluated by van Paasschen et al. [28]. The goal of this study was to relate clinically relevant improvement and differences in brain activity. A passive control group with no treatment as well as an active control group with relaxation therapy was used. In 8 weekly sessions, personalized goals concerning memory were targeted in the cognitive rehabilitation. The main outcome measure was the Canadian Occupational Performance Measure (COPM) that rated the satisfaction and performance of participants on several goals with respect to daily living. Brain activity was studied with fMRI [28].

Whereas most studies selected fMRI to study brain activation, Spironelli et al. [36] used Event Related Potentials (ERP) to evaluate possible changes in brain functioning after cognitive training in 11 people with mild to moderate AD. Their intervention aimed to stimulate different cognitive domains based on everyday activities and exercises. The 11 matched healthy controls underwent one experimental ERP session to serve as a reference for possible altered response patterns. The intervention success was also evaluated with neuropsychological tests and questionnaires of everyday activities [36].

Two studies used PET data as the neurobiological outcome. One study was a single case design on the effect of eight weekly reminiscence sessions on activities of daily living, cognition, volition, vitality, behavioural problems, well-being, caregiver burden, and PET data. A comprehensive geriatric assessment was also administered six months after intervention [35]. The other study provided 11 VaD patients with recreational rehabilitation. They used PET to study blood flow differences after intervention. The group of 11 patients was divided into responders (improved more than 3 points on the MMSE) and nonresponders [34].

#### 3.3.2. Findings

The eight studies that investigated cognitive intervention effects on brain functioning of people suffering from dementia differed in design, type of intervention, duration of the intervention, type of neurobiological and behavioural outcomes, and the presence of follow-up measures (Table 3). Despite those differences, all studies found neurobiological changes after intervention.

In the most recent RCT study, an increased activity in the superior temporal gyrus bilaterally, the thalamus and the right lentiform nucleus was seen after a multidimensional stimulation program. The intervention group also showed clinical relevant improvement in neuropsychiatric symptoms, language, and memory scales of a cognitive screening. There is a significant correlation between the magnitude of increased activity in the left superior temporal gyrus, precuneus and left thalamus, and the change in cognitive screening performance. The improvement in these scales of the cognitive screening is preserved at 22 weeks afte intervention [29].

Another multidimensional intervention found evidence that participants with AD showed decreased activity in two clusters (lingual gyrus, left inferior temporal gyrus), whereas the active control group showed decreased activity in a larger network prefrontal, parieto-occipital and parieto-temporal. However, there were no significant changes in the behavioural outcomes [27].

Another RCT study showed that tailored cognitive rehabilitation induced increased activity in prefrontal areas and insula bilaterally, whereas both active and waiting list control groups showed decreased activity in those areas. The intervention group improved on satisfaction and performance of individual goals; both control groups showed no improvement [28].

A RCT study that evaluated the effect of reminiscence with reality orientation found that intervention related increased activity in the anterior cingulate correlated significantly with improvement in social and communication scales [26].

A single case study on the effect of reminiscence reported increased activity in frontal areas, precuneus and posterior cingulate gyrus after intervention. The participant also improved on measures of cognition, vitality, volition, and daily life activities [35].

In a RCT study hat evaluated the effect of four different interventions, global EEG power increased in both intervention groups that combined cognitive training with a dietary supplement or a symptomatic drug for dementia. There were no significant changes in brain metabolism after intervention in predetermined target regions. Additional regions of interest were analysed; this revealed an increase in metabolism in the visual association area during functional activation for the cognitive training and phosphatidylserine group. For this intervention group there was also an increase in resting state metabolism in temporal regions, but only for the participants in this particular intervention group that had initial metabolism values below 90% of normal in the temporal region. There were also some behavioural benefits for this intervention group. In weeks 8 and 16, the group that received cognitive training and phosphatidylserine scored significantly better on orientation than the other intervention groups. This effect was no longer present at the end of the intervention [25].

An increased amplitude of the recognition potential for high frequency words on the left sides of posterior regions after cognitive training was demonstrated in an ERP study. Most behavioural outcomes fail to show a significant intervention effect, despite a marginally significant improvement on verbal reasoning [36].

Responders of a recreational rehabilitation intervention showed decreased activity in frontal areas after intervention. The nonresponders showed activity decreases in a large network of brain areas after intervention [34].

Thus, most studies found an increase in brain activation after intervention or less decrease in activation versus the control group. One study reported that the responders in their intervention showed a significant decrease in cerebral blood flow in the frontal regions, whereas nonresponders showed a decrease in a larger network of brain areas. Their study, however, is the least quality of all the included studies [34]. According to one study [25], the changes in neuropsychological measurements and brain activity were temporary and disappeared at the end of the six-month intervention. Two studies however demonstrated a behavioural intervention success of 22 weeks and six months after intervention. Both studies did not follow up with the neurobiological changes [29, 35]. The three most recent studies [28, 29, 36] and the study of Tanaka and colleagues [35] tried to link clinical improvement to changes in brain activation by using behavioural outcomes that were not only cognitive tasks but more related to daily functioning, individual goals, subjective complaints, or social skills. Baglio and colleagues took this one step further to statistically relate the brain activation changes to the behavioural outcomes [29].

### 3.4. Methodological Quality

The selected 19 studies were quite heterogeneous in terms of design, sample size, population, intervention methods, and neurobiological outcome measurements. Therefore, it was decided to not statistically pool the data to perform a quantitative meta-analysis. Eight studies were comprised of patients with a diagnosis of dementia; five studies involved patients with Mild Cognitive Impairment; and six studies included healthy elderly. Of the selected studies there were 13 randomized controlled trials, 3 prospective studies, 1 study using a within-subjects design, and 2 single case studies.

The overall methodological quality of the included studies varied. For the RCTs, the overall score of the methodological quality varied from 3 to 9 (maximum 9) with a level of evidence ranging from A2 to B according to the CBO (Table 1). The overall score for the methodological quality of the observational studies varied from 3 to 9 (maximum 9). The CBO level of evidence ranged from B to C (Table 2).

Six of the 13 RCT studies lacked an intention to treat analysis. Randomization was blind in only six of the 13 RCT studies. In more than half of the studies the outcome assessor was not blind to the treatment condition. In the observational studies, almost none of the studies had blinded outcome assessors and almost every study had a selection bias.

A hierarchy of quality was composed based on the design and methodology. With regard to the studies that focused on healthy elderly, the study of Belleville et al. [23] had the highest methodological quality. For the studies that comprise patients with MCI, the studies of Belleville and Bherer [15] and Erickson et al. [21] had the highest methodological quality. The study with the highest methodological quality that involved patients with dementia was that of Baglio et al. [29]. Akanuma et al. [26] was limited in the informative value due to a lack of detailed methods and results.

## 4. Discussion

In this paper, the literature was reviewed to investigate whether cognitive interventions in elderly lead to changes in brain activation suggestive of neural plasticity even in damaged neural systems. The methodological quality of the 19 studies was rated according to the guidelines of the Dutch Institute for Health Care Improvement (CBO, http://www.cbo.nl/).

The results illustrate that all studies, conducted in diverse populations from healthy elderly to patients with dementia, show changes in brain activation post intervention. The methodological quality of the studies varied with the CBO level of evidence ranging from A2 to C (Tables 1 and 2).

All four studies in healthy elderly found brain activation differences after intervention. Two studies found increases in brain activation post intervention, primarily in the occipital parietal cortex and retrosplenial cortex [30] as well as in the hippocampus [18]. Post hoc only the older participants that improved on a memory test showed these brain activity increases [30]. However, this could not be documented by more clinical outcomes due to a lack of such outcomes or a fail to link brain activation changes to clinically meaningful improvement.

On the contrary, another study found a decrease after intervention in the dorsolateral brain activity [20]. The relationship between this decreased activity dorsolateral and clinically relevant improvement was not evident. Only an improvement in verbal fluency was found, but no significant intervention effects were found on the subjective measurements of self-awareness of memory performance and other cognitive tasks [20]. Three studies successfully linked intervention-related performance gains to brain activation changes by statistical analyses. One study found performance-related brain activity decreases in the cortical regions known to be involved in cognitive functions targeted by the intervention and performance related brain activity increases in subcortical areas [22]. Another study provided evidence that training induced dual task performance improvement was related to increased activity in the left ventral prefrontal cortex and decreased activity in the dorsolateral prefrontal cortex. Compared with an adult sample, age related differences in brain activity were reduced after intervention [21]. The study of Belleville and colleagues [23] concluded that the type of intervention influenced the loci and type of brain activity. A better performance in single tasking was correlated with decreased activity in the right inferior and middle frontal gyrus. An improved ability to modulate attention according to task instruction was correlated with a greater activity in Brodmann area 10 [23].

Two studies argued that the decreases in brain activity could be explained by an increased cognitive efficiency, thus demanding less effort [20, 22]. The increase in subcortical brain activity was, according to these authors, evidence that the performance was becoming less executively demanding and more proceduralized as the training proceeded [22]. This is in line with the theory of Bartrés-Faz and Arenaza-Urquijo on cognitive efficiency [19]. In addition, the study with the highest methodological quality highlighted the importance of the type of intervention format. Intervention format has effect on the loci and type of brain activation changes after intervention. They refer to the framework of Lorden and their own theoretical framework named INTERACTIVE [21]. These models state that repeated practice would lead to decreased activity in the brain areas involved in the task, indicating increased cognitive efficiency. Interventions that aim to learn participants new strategies will lead to increased activity in alternative networks involved in learning those strategies [21].

Another study interpreted the performance related brain activity pattern of increased activity in the left ventral prefrontal cortex and decreased activity in the dorsolateral prefrontal cortex, as evidence conflicting with views of compensation related reduced brain activity asymmetry. While brain activity differences between adults and older adults were significantly reduced after training, they interpreted this as evidence that cognitive training can modulate age related patterns of brain activity [23].

All of the reviewed studies of cognitive interventions in MCI patients found neurobiological changes in brain activation after intervention despite differences in methodology. There was mostly an increase in brain activation, in areas typically involved in intervention related cognitive processes. This was seen as well as activation of the default network. In several studies, even normalisation of the pattern of brain activation after intervention was claimed. The study of Rosen et al. had the highest methodological quality in this review and showed that even brain structures known to be injured in patients with MCI such as the hippocampus retained sufficient brain plasticity to benefit from cognitive interventions [16]. This activation of the default network and hippocampus is in line with Bartres' assumption that, in pathological aging, the cognitive reserve enhances the recruitment of compensating brain networks particularly the frontal areas, hippocampus, and the precentral gyrus [19]. Cognitive behavioural interventions might increase this cognitive reserve [4, 8]. Functionally relevant clinical improvement has been given little attention in these studies. A statistical approach to the relationship between intervention success on behavioural measures and brain activation changes was only made by one study [26]. Long-term follow-up measures to determine the stability of the intervention success were not included.

Studies in dementia patients also found neurobiological changes after intervention. Most of the studies found an increase in brain activation or a diminished decrease in activation after intervention. The study of Förster et al. [27] was the only one that found a decrease in activation post intervention, but the construct of this study has poor methodology with little detailed information about the methods and results. According to one study that used a six-month intervention, the increased brain activity and improvement on neuropsychological tests were temporary in persons with AD and then disappear over time [35]. Other studies demonstrated a preserved improvement in the clinical outcomes such as cognitive tasks, daily life activities, and vitality at 22 weeks and six months after intervention. However, they did not follow up the neurobiological outcomes [26, 32]. Thus, it remained unclear if the changes in brain functioning were temporary. The study with the highest methodological quality correlated improvement in social and communication scales with increased activity in the anterior cingulate, an area known to be involved in social behaviour [26].

One study excluded [27]; all of the authors interpreted their findings as evidence for brain plasticity. Some even stated that cognitive interventions activate compensating brain networks in pathological ageing and could possibly restore brain activation. However, the stability of this effect remains unclear.

Overall, these results suggest that cognitive interventions lead to neurobiological changes even in potentially damaged neural systems. However, the interpretation of changes in activation patterns is complicated. For instance, a decrease in brain activation can indicate increased cognitive efficiency as suggested in the cognitive reserve hypotheses of Bartrés-Faz and Arenaza-Urquijo [19] and the learning phases model of Doyon in Lustig et al. [37]. On the other hand, a decrease in brain activation might reflect exhaustion of neural resources accompanied by a decline in clinical performance as suggested by the CRUNCH model [37].

Moreover, the type of intervention would influence the loci and type of activation changes after intervention [21].

The complexity of interpreting these neurobiological data underlines the importance of including clinical measurements to gain insight into the clinical relevance of neurobiological changes. Unfortunately, in several studies, this information is lacking or neurobiological data is interpreted as evidence for plasticity/restoration of function in the absence of a demonstrated intervention success on clinical outcomes. In the more recent studies, there is increased attention for the clinical relevance of neurobiological changes with promising results. In five studies [21–23, 26, 32] performance gains were linked to brain activation. Four studies used correlations to correlate improvement on a dementia screening test [22], social and communication scales [26] and changes in performance on different attentional tasks [21, 23] to relevant changes in brain activation. A fourth study used the maximum gain score on working memory tasks as a covariate in statistical analysis [32].

These studies focus on relating performance improvement to brain activity changes. However, the transfer of these performance gains to untrained tasks and more importantly to daily life functioning is a great issue [21].

The heterogeneity in populations, outcome measures and interventions as well as the small sample sizes and relatively large amount of case studies further complicate the comparability of the findings between studies.

We recommend that future studies should include measures of clinically meaningful improvement as well as long-term follow-up data to evaluate the stability of the effects. The influence of task demands, premorbid cognitive reserve, and learning phases on brain activation should be considered to increase comparability between studies. The results thus far indicate that the elderly show changes in brain activation after cognitive interventions. However, the exact interpretation and stability of these changes remain unclear just like to what extent cognitive interventions are effective to reach the ultimate goal: to delay conversion to or prevent progression of dementia.

## Figures and Tables

**Table 1:** Methodological quality of randomized controlled trials.

RCT	The healthy elderly						MCI			Dementia				
	Valenzuela et al. 2003 [18]	Small et al. 2006 [20]	Erickson et al. 2007 [21]	Brehmer et al. 2011 [22]	Belleville et al. 2014 [23]		Rosen et al. 2011 [16]	Hampstead et al. 2012 [24]		Heiss et al. 1994 [25]	Akanuma et al. 2011 [26]	Förster et al. 2011 [27]	van Paasschen et al. 2013 [28]	Baglio et al. 2014 [29]
Randomized	1	1	1	1	1		1	1		1	1	1	1	1
Blinded randomization	0	0	1	0	1		1	1		0	1	0	0	1
Blinded participants	0	0	0	1	1		1	1		0	0	0	0	0
Blinded outcome assessors	0	0	0	0	0		1	0		0	1	1	0	1
Baseline comparability	0	1	1	1	1		1	1		1	1	1	1	1
Loss to follow	1	1	1	1	1		1	1		1	1	0	1	1
Intention-to-treat analysis	0	1	0	0	0		1	1		0	1	1	1	0
Comparability intervention	0	0	0	1	1		1	1		1	0	1	0	1
Validity and applicability	1	1	1	1	1		1	1		1	1	1	1	1

Total	3	5	5	6	7		9	8		5	7	6	5	6
CBO classification	B	B	B	B	A2		A2	B		B	B	B	B	B

The CBO classification reflects the level of evidence. A1 is a systematic review of at least two independent randomized double blind studies of sufficient quality and size. A2 is a randomized double blind study of sufficient quality and size. B is a comparative study, which does not meet all the criteria of an A2 study. C is a noncomparative study and D is the opinion of experts (http://www.cbo.nl/).

**Table 2:** Methodological quality of observational studies.

Observational studies	The healthy elderly	MCI			Dementia		
	Nyberg et al. 2003 [30]	Clare et al. 2009 [31]	Hampstead 2011 [32]	Belleville et al. 2011 [33]	Nagaya et al. 2005 [34]	Tanaka et al. 2007 [35]	Spironelli et al. 2013 [36]
Definition study population	0	1	1	1	0	1	1
Selection bias	0	0	0	1	0	0	0
Intervention description and allocation	1	1	1	1	1	1	1
Outcome definition	1	1	1	1	1	1	1
Blinded outcome assessments	0	0	0	1	0	0	0
Completeness dataset/follow-up	0	1	1	1	0	1	1
Loss to follow	0	1	1	1	1	1	1
Confounders	1	0	0	1	0	0	0
Validity and applicability	0	1	0	1	0	0	1

Total	3	6	5	9	3	5	6
CBO classification	C	C	C	B	C	C	C

The CBO classification reflects the level of evidence. A1 is a systematic review of at least two independent randomized double blind studies of sufficient quality and size. A2 is a randomized double blind study of sufficient quality and size. B is a comparative study, which does not meet all the criteria of an A2 study. C is a noncomparative study and D is the opinion of experts (http://www.cbo.nl/).

**Table 3:** Main characteristics of selected studies.

Study	Design Duration Long-term follow- up *n*	Intervention	Result neurobiological outcomes	Result behavioural outcomes
Healthy	The elderly			

Valenzuela et al. (2003) [18]	RCT 5 weeks No follow-up *n* = 20	Method of loci	Increased creatine and choline in hippocampus	Improvement in reproduction memory task No effect on depression or anxiety scores

Nyberg et al. (2003) [30]	Prospective cohort 1 day No follow-up *n* = 24	Method of loci	**Acquisition**: no group differences **Use phase**: adults and improved elderly increased activity in intervention related areas	8 of 16 older persons no improvement in memory task (the unimproved elderly). 8 of 16 older persons and all 8 adults improved in memory task

Small et al. (2006) [20]	RCT 14 days No follow-up *n* = 17	Multicomponent health promotion	Intervention group: decreased activity prefrontal cortex	Better verbal fluency in intervention group no significant effect on memory task and subjective memory ability

Erickson et al. (2007) [21]	RCT 2-3 weeks No follow-up *n* = 65	Attentional training	Improvement in dual task performance is correlated with increased activity in left ventral prefrontal cortex and decreased activity in the dorsolateral prefrontal cortex	Both reaction time and accuracy improved most in the dual task intervention group Young and old adults showed the same degree of performance improvement after intervention.

Brehmer et al. (2011) [22]	RCT 5 weeks No follow-up *n* = 24	Adaptive working memory training. Control group: low level fixed working memory training	All participants decreased brain activity Participants who profit the most showed the largest decreases in intervention related brain areas and largest increase in caudate	Both groups Improved in span board backward, digit span backward, PASAT, RAVLT No intervention related performance gains for in scanner task Intervention group showed training related improvement in span board backward task and PASAT compared to controls

Belleville et al. (2014) [23]	RCT 3 weeks No follow-up *n* = 46	Attentional training	Better performance in single tasking correlated with decreased activity in right inferior and middle frontal gyrus In the strategic control of attention condition, a better ability to modulate attention according to task instruction correlated with increased activity in Brodmann area 10	All intervention groups improved in reaction time and accuracy for alphanumeric task, no effect for visual detection task Both dual task conditions better performance dual tasking compared to single task intervention group Strategic control of attention condition significant effect of task instruction. They were able to modify attention according to instruction.

Mild	Cognitive	Impairment		

Clare et al. (2009) [31]	Single case study 8 weeks No follow-up *n* = 1	Goal oriented cognitive intervention	**Encoding**: increased activity in intervention related brain areas, decreased activity higher visual areas, and frontal areas **Recognition**: increased activity in intervention related brain areas, decreased activity higher visual areas, and frontal areas	Better subjective memory performance, memory satisfaction

Hampstead et al. (2011) [32]	Multiple single cases 2 weeks No follow-up *n* = 6	Face-name association learning	**Encoding**: increased activity in default network **Effective connectivity changes**: increased connectivity	Significant improvement in performance trained and untrained memory task

Belleville et al. (2011) [33]	Case control 6 weeks No follow-up *n* = 15	Episodic memory training	**Encoding healthy elderly**: decreased activity in brain areas related to intervention. **Encoding MCI**: increased activity in brain areas related to intervention **Retrieval healthy elderly and MCI**: increased activity new brain areas and accumulated activity in specialized areas both related to intervention.	Both groups improved on a memory test

Rosen et al. (2011) [16]	RCT 2 months No follow-up *n* = 12	Auditory processing training	Increased activity hippocampus in intervention group decreased activity hippocampus in control group	Intervention group improved in memory test and training tasks

Hampstead et al. (2012) [24]	RCT 2 weeks No follow-up *n* = 34	Mnemonic training	**Encoding MCI**: increased activity left hippocampal body **Retrieval MCI**: increased activity hippocampal body and tail bilaterally **Healthy controls**: decreased activity right hippocampal body	MCI group and healthy controls improved in encoding and retrieving trained object locations. No intervention effect for untrained object locations.

Dementia				

Heiss et al. (1994) [25]	RCT 6 months No follow-up *n* = 80	(1) Social support (2) Cognitive training (3) Cognitive training + pyritinol (4) Cognitive training + phosphatidylserine	**EEG**: increased global power gr 3 + 4 Decreased delta power gr 4 **PET**: significant correlation MMSE score and glucose metabolism tempero-parietal cortex. Gr. 4 increased activity primary visual cortex during intervention, but not at the end of the intervention	Gr 4 more responders and significant higher scores on orientation than gr 1 + 2 in week 8 + 16. At the end of the intervention (6 months) there were no differences.

Nagaya et al. (2005) [34]	Within subjects Unknown No follow-up *n* = 11	Recreational rehabilitation	**Responders**: decreased activity frontal regions **Non responders**: decreased activity all regions	Responders: improved 3 MMSE points

Tanaka et al. (2007) [35]	Single case 2 months Follow-up for 6 months *n* = 1	Reminiscence	increased activity frontal areas, postcingulate gyrus, and precuneus	Improvement in cognition, vitality, volition, and daily life activities

Förster et al. (2011) [27]	RCT 6 months No follow-up *n* = 36	Multipurpose	**MCI controls**: decreased activity in brain regions typically impaired in AD **MCI intervention**: no declines **AD controls**: decreased activity in brain regions typically impaired in AD **AD intervention**: decreased activity in 2 clusters; lingual gyrus, left inferior temporal gyrus	No changes

Akanuma et al. (2011) [26]	RCT 3 months No follow-up *n* = 24	Reminiscence with reality orientation	**Intervention group**: increased activity anterior cingulate bilateral, left inferior temporal cortex. Correlation between increased act anterior cingulate and improvement social and communication scales brse	**Intervention group**: improved in social and communication scales (brse)

Spironelli et al. (2013) [36]	Observational study 5 weeks No follow-up *n* = 11	Cognitive training	The amplitudes of the recognition potential (negative potential) were significantly increased on the left sides of posterior regions for high frequency words	A marginally significant improvement on the verbal reasoning score No significant treatment effect on the dementia screening tests, four out of five cognitive tasks, and the independent functioning questionnaires.

van Paasschen et al. (2013) [28]	RCT 8 weeks No follow-up *n* = 19	Tailored cognitive rehabilitation	*Intervention group* **Encoding**: no significant changes. **Recognition**: significant increased activity bilateral prefrontal areas and the bilateral insula *Control group* **Recognition**: decreased activity in bilateral prefrontal areas and the bilateral insula	Intervention group improved on satisfaction and performance of individual goals (COPM) No treatment effect on the in scanner face-name association task

Baglio et al. (2014) [29]	RCT 10 weeks Follow-up for 22 weeks *n* = 60, *n* = 30 Follow-up	Multidimensional stimulation program	**Postintervention**: increased activity bilateral superior temporal gyrus (right > left), right lentiform nucleus, and thalamus	Intervention group showed clinical relevant improvement in NPI, language, and memory scales of ADAS-cog No significant change in functional status or physical well-being **Long-term follow-up**: improvement in the language and memory scales of the ADAS-cog is preserved

Legenda:

qol: quality of life.

MMSE: minimental state examination.

ADAS cog: dementia screening test.

BRSE: scale for social and communication skills.

GDS: geriatric depression scale.
